# Exploring *Myrciaria dubia* liquid extract as a potential semen extender for breeding roosters

**DOI:** 10.1590/1984-3143-AR2024-0020

**Published:** 2024-10-04

**Authors:** Marcia Lorena Monteiro da Silva, João Paulo Ferreira Rufino, Brenda de Meireles Lima, Marco Antonio de Freitas Mendonça, Francisco Alberto de Lima Chaves, Roseane Pinto Martins de Oliveira, Pedro de Queiroz Costa, Paulo Cesar Machado Andrade

**Affiliations:** 1 Faculdade de Ciências Agrárias, Universidade Federal do Amazonas, Manaus, AM, Brasil

**Keywords:** camu-camu, diluent, Myrciaria dubia, semen extender, semen quality

## Abstract

The current investigation aimed to explore the effects of *Myrciaria dubia* liquid extract (MDLE) as the primary component of an extender for breeder rooster semen over different periods at room temperature. Fifteen breeder roosters (40 weeks of age, average body weight of 2.05±0.12) with confirmed fertility were used. Employing a factorial design (3x4), the treatments consisted of semen *in natura* and two semen extenders (an experimental based on MDLE and a commercial) subjected to four periods at room temperature post-collection (5, 10, 15 and 20 minutes) with four replicates (tubes) each. All variables evaluated in this study yielding significant results (p<0.05). Analyzed individually, the experimental extender based on MDLE exhibited a linear reduction (p<0.05) in motility and vigor results, while it caused an increase in pH values and percentages of sperm defects evaluated. When compared with semen *in natura* and commercial extender, the efficiency of MDLE as a semen extender was inferior to that observed with the commercial extender and similar to the results observed with semen *in natura*. Nonetheless, the experimental extender based on MDLE yielded satisfactory results for up to 15 minutes of storage time. In conclusion, MDLE can be considered as an alternative for composing a roosters’ semen extender, maintaining sperm characteristics within acceptable limits for up to 15 minutes at room temperature. However, this experimental extender demonstrated lower efficiency than the commercial extender in maintaining the sperm quality at room temperature across all periods tested.

## Introduction

Modern biotechnology applied in animal science focuses on investigating techniques for collecting, evaluating, and preserving semen for breeding and genetic improvement (Freitas et al., 2018). In poultry science, this technology can contribute to the development of more efficient procedures for preserving rooster semen for longer periods, utilizing artificial insemination for breeder selection, and monitoring the performance of high-quality progeny compared to the breeders themselves (Rutz et al., 2007; Bongalhardo et al., 2009; Rufino et al., 2014; Feijó et al., 2016).

For poultry farms, the dilution and preservation of semen for extended periods offer valuable support in breeder selection programs (Massip et al., 2004; Morais et al., 2012). This is particularly crucial because rooster semen is highly concentrated, yielding a low volume compared to other farm animal species. Additionally, rooster semen has a limited lifespan at room temperature, typically lasting up to 20 minutes (Rutz et al., 2007; Partyka et al., 2012; Bongalhardo, 2013; Freitas et al., 2018).

In the poultry industry, a wide array of extenders (or diluents), preservatives, protectants, and protocols have been extensively tested and are available to enhance the performance of rooster semen (Rutz et al., 2007; Silva and Guerra, 2011; Lavor and Câmara, 2012). However, owing to the inherent fragility of rooster semen when handled at room temperature and its unique cellular structural characteristics, continuous efforts are underway to explore new diluents and preservatives. The aim is to preserve the essential properties of natural seminal fluid, ensuring stability and longevity for sperm while offering alternatives to chemical-laden and costly products (Purdy et al., 2009; Bongalhardo, 2013).

Among the potential Amazonian products that could serve as alternative extenders for rooster semen, the extract of *Myrciaria dubia* (commonly known as *camu-camu* in Brazil) stands out for its intriguing composition. This extract is particularly rich in bioactive compounds such as ascorbic acid, phenolic compounds, and β-carotene (Chirinos et al., 2010). Akachi et al. (2010) also noted significant levels of polyphenols, fructose, glucose, anthocyanins, potassium, calcium, vitamin A, starch, pectin, phosphorus, and antioxidant compounds in *Myrciaria dubia* extract.

Based on this premise, we hypothesized that utilizing *Myrciaria dubia* liquid extract (MDLE) as the primary component of an extender for breeder rooster semen, given its nutrient-rich composition, could potentially enhance the quality of breeder rooster semen for prolonged periods at room temperature. Moreover, the decision to test the efficiency of MDLE at room temperature was made to simulate practical and realistic conditions that would be encountered in a typical poultry farm environment (Rutz et al., 2007; Lavor and Câmara, 2012; Partyka and Nizanski, 2022).

Although the literature indicates that storing semen at temperatures between 4 and 5 °C is a more efficient method to reduce the metabolism of sperm and maintain its viability for a relatively longer period compared to samples handled at room temperature, this method is very costly (Blesbois, 2007; Partyka and Nizanski, 2022). It requires infrastructure and equipment that allow both prolonged storage and the possibility of transporting it during handling for insemination, making its use unfeasible for many poultry producers (Blesbois, 2007; Rutz et al., 2007). Therefore, testing this at room temperature ensures the findings are directly applicable and relevant to scenarios commonly faced by the producers, where the ideal room temperature in poultry farms typically ranging between 20-25 °C (Blesbois, 2007; Partyka and Nizanski, 2022). Thus, the objective of this study was to explore the effects of MDLE as the primary component of an extender for breeder rooster semen over different periods at room temperature.

## Methods

The current experiment was conducted at the Research Poultry Farm of the Federal University of Amazonas, located on the university campus in the city of Manaus, Amazonas State, Brazil. All experimental procedures were approved by the institutional ethics committee (protocol number 011/2021) of the Federal University of Amazonas, Brazil.

### Obtaining *Myrciaria dubia* liquid extract

The fruits of *Myriciaria dubia* were obtained from cultivation at the Brazilian Agricultural Research Corporation (Embrapa Western Amazon), located on AM 010 highway, Km 29, Manaus-Itacoatiara. The seeds were separated from the husk and pulp of the fruit, then subjected to drying in a forced air circulation oven at 40 °C for 5 days, followed by grinding in a knife mill. The resulting plant material was stored in 1-liter plastic containers at room temperature. For obtaining the liquid extract of *Myriciaria dubia*, the methods described by Carmo et al. (2019) were used. Seventy-five g of the plant material were weighed for 1000ml of 20% (v/v) hydroalcoholic solution. Subsequently, the hydroalcoholic solution was poured over the plant material, and the resulting mixture was macerated for 48 hours with constant agitation at room temperature. After the 48-hour maceration period, the liquid extract underwent a standard filtration process using gauze and cotton, followed immediately by vacuum filtration. The resulting MDLE had a concentration of 7.5% (m/v).

A sample of the MDLE was used to determine its phenolic composition. The total phenolic content was quantified using the Prussian Blue method described by Margraf et al. (2015), and the results were expressed as mg of gallic acid equivalent (GAE) per 100 g of seed (mg GAE/100 g). Total condensed tannins were estimated using the vanillin-H_2_SO_4_ method according to Horszwald and Andlauer (2011), with the values expressed as mg of (+)-catechin equivalent per 100 g (mg CTE/100 g). Phenolic compounds of different classes (phenolic acids, flavonoids, ellagic acid, and stilbene) were determined by high-performance liquid chromatography (HPLC), Shimadzu LC-20 T, equipped with DAD (diode detector array) and fluorescence detectors, a degasser system, auto sampler, and oven column, following the method described by Fidelis et al. (2018).

The average total phenolic content of MDLE was found to be 176 mg GAE/100 g, which includes rosmarinic acid, 2,4-dihydroxybenzoic acid, ellagic acid, cyanidin-3-glucoside, methylvescalagin, trans-resveratrol, and quercetin. Similarly, Fidelis et al. (2018) analyzed the bioactive compounds of *Myrciaria dubia* seed coat and identified the presence of these same compounds. Regarding the condensed tannins content, a concentration of 264 mg CTE/100 g was observed. Notably, caffeic acid and 2,5-Dihydroxybenzoic acid were also detected in the MDLE.

### Roosters and experimental design

The roosters utilized in this study belonged to a breed known as PR, which is a hybrid resulting from the crossing of Label Rouge roosters and Rhode Island Red chickens in a 50/50 genetic proportion. Label Rouge roosters were selected for their great body weight, feed efficiency, feed intake, carcass traits, viability, fertility and hatchability, and reduction of abdominal fat and metabolic diseases. On the other hand, Rhode Island Red chickens were chosen for their exceptional egg production, egg quality, feed efficiency, viability, sexual maturity, fertility, hatchability, and body conformation. It was observed that these breeds possess divergent characteristics. Thus, through the crossbreeding of these breeds, a population of PR breeders displaying significant hybrid vigor was obtained.

Breeder roosters (n = 15; 40 weeks of age; average body weight of 2.05±0.12) identified and previously verified for fertility were selected for this study. They were housed in a commercial aviary with a density of 1 bird/m^2^, receiving a daily feed allowance of 115 g/bird/day of balanced diets calculated meeting the requirements described by Rostagno et al. (2017), and having access to water *ad libitum*. The experimental design followed a factorial arrangement (3x4), with treatments consisting of semen *in natura* and semen diluted in two types of extenders (experimental and commercial), applied at four different periods (5, 10, 15 and 20 minutes) at room temperature, each with four replicates (tubes) each.

### Semen collection and extenders preparation

Individual semen collections were conducted following the methodology described by Burrows and Quinn (1937), which involved applying abdominal massage to the back and sides of the cloaca of roosters. During collection, semen from each rooster was collected into graduated tubes. The average semen volume obtained per rooster was 0.8 ml. It is noteworthy that all 15 roosters utilized in this study exhibited semen production.

All semen samples collected were handled in a controlled environment at 26 ºC (78,8 ºF) and 55% relative humidity. These samples were immediately pooled to homogenize the ejaculates and ensure equal distribution of sperm. The pooled semen was then divided into 12 graduated Eppendorf tubes of 1 mL each, according to predefined treatments: 4 tubes containing only semen *in natura*, 4 tubes containing a commercial extender, and 4 tubes containing an experimental extender.

The tubes containing semen *in natura* were not diluted in any extender, and being handled under environmental conditions and analyzed using methods similar to those of the diluted samples. The tubes containing extender (commercial or experimental) were diluted at a ratio of 2 to 1 (2 parts of extender to 1 part of semen) following the recommendations of the Brazilian College of Animal Reproduction (2013).

The commercial extender utilized was the Beltsville Poultry Semen Extender (BPSE; Continental Plastic Corporation, Delavan, WI), which is a standard extender commonly used for avian semen preservation (Lotfi et al., 2017; Siari et al., 2021). The composition of this commercial extender included sodium glutamate (8.67 g/l), dipotassium phosphate (7.59 g/l), sodium acetate (3.2 g/l), fructose (5 g/l), potassium citrate (0.64 g/l), n-tris (hydroxymethyl) methyl 1-2 amino ethane sulfonic acid (TES; 3.2 g/l), monopotassium phosphate (0.7 g/l) and magnesium chloride (0.34 g/l). Osmolarity and pH were set at 310 mOsm/kg and 7.1, respectively.

Preparation of the experimental extender was performed as described by Marco-Jiménez et al. (2004). Briefly, the experimental extender was prepared by combining the MDLE previously obtained and soy lecithin in a ratio of 3 to 1 (3 parts of MDLE to 1 part of soy lecithin) in a controlled environment at 26 ºC (78,8 ºF) and 55% relative humidity, with this mixture being gently agitated for approximately 30 seconds. Given the natural acid pH of MDLE, the pH of the experimental extender was adjusted using Dibasic Sodium Phosphate to levels recommended by the Brazilian College of Animal Reproduction (2013). Soy lecithin (Adicel Ind. e Com. LTDA^©^, Belo Horizonte, Brazil) was combined with the MDLE to synergistically aid in the structural and functional maintenance of the plasma membrane of rooster sperm, thereby enhancing its preservative effect on the sperm (Silva et al., 2019).

### Studied parameters

Both the semen *in natura* and diluted samples at their respective storage periods had a droplet of semen solution placed between a slide and coverslip for evaluation under light microscopy at 400x magnification. Each sample was assessed for motility (percentage of motile sperm during analysis, ranging from 0 to 100%), vigor (straight and uniform movement of sperm on a scale from 0 to 5) and pH (determined using a pH meter coupled with a fine-tipped probe directly in the semen samples). These semen characteristics were evaluated using a Nikon Eclipse E-50i microscope (Tokyo, Japan) connected to a camera and computer-assisted semen analysis (Microptic S.L., Barcelona, Spain). This evaluation followed the methods and reference values described by the Brazilian College of Animal Reproduction (2013).

Afterward, other slides were prepared using SpermBlue staining, following the method described by van der Horst and Maree (2010). A thin semen smear was spread on a microscope slide heated to approximately 36 °C. Once the smears had dried, they were stained with SpermBlue for approximately 15 minutes. Subsequently, the slides were washed with distilled water and allowed to dry at room temperature.

On each slide, the morphological structure of 200 cells was assessed. The number of sperm cells with normal structure and the number of morphologically abnormal sperm cells were determined, distinguishing forms with major and minor abnormalities according to the classification described by Blom (1981) and the Brazilian College of Animal Reproduction (2013).

### Statistical analyses

The collected data were analyzed in two stages: firstly, only the data collected using MDLE as an extender for roosters’ semen to evaluate its efficiency in different periods; and secondly, comparing the efficiency of the use of MDLE as an extender for roosters’ semen with semen *in natura* and the commercial extender. Before conducting statistical analysis, all data were assessed for normality and transformed if necessary. Subsequently, one-way ANOVA was performed on all data using R software (version 4.1.3), following the procedures reported by Logan (2010).

In the first stage, polynomial regression was applied to analyze the influence of the independent variable on dependent variables in a linear (Y = a + bx) or quadratic model (Y = ax^2^ + bx + c) (Chatterjee and Hadi, 2006; Logan, 2010). The math model was adjusted according to the influence of each independent variable on the dependent variable analyzed (Dormann et al., 2013). The results are presented as means, and the significant level for differences was set as p<0.05. Additionally, R-squared values were considered as a factor to indicate best model fit (Chatterjee and Hadi, 2006; Dormann et al., 2013).

In the second stage, Tukey’s honestly significant difference test was utilized to test the significant differences among the mean values and compare the efficiency of MDLE as an extender for roosters’ semen relative to semen *in natura* and the commercial extender. The results are presented as means, and the significance level for differences was set as p<0.05.

## Results

The initial semen samples collected were utilized for preliminary quality analyses of the pure ejaculate following the guidelines outlined by the Brazilian College of Animal Reproduction (2013). These results are independent of extender action but directly influence their responses. Upon analysis, the mean sperm concentration of the studied animals, after dilution in formalin-saline solution and counting in the Neubauer chamber, ranged from 3.5 to 3.8x10^6^ spermatozoa/ml. Mass movement was observed in all sperm samples collected, and similarly, the ejaculate appeared white and milky in all samples. The pH of the samples varied between 6.9 and 7.3.

In the initial stage of analysis, all evaluated variables (refer to Table 1) exhibited a significant effect (p<0.05). The results for motility and vigor showed a negative linear trend with increasing exposure period of sperm to the experimental extender based on MDLE. Conversely, the percentages of sperm defects, both total and specifics, demonstrated a positive linear trend with increasing exposure period to the experimental extender. These findings suggested a notable and substantial decline in sperm quality as the duration of exposure to the experimental extender based on MDLE increased.

**Table 1:** Isolated effects of an experimental extender based on *Myrciaria dubia* liquid extract for roosters’ semen stored for 20 minutes.1

**Variables**	**Storage periods (minutes)**				**CV** **2** **, %**	**p-value** **3**	**Model** **4**	**R^2^**
	**5**	**10**	**15**	**20**				
Motility, %	66.50±8.10	60.64±7.28	56.30±15.50	32.65±14.32	5.67	0.01	Y = 80.4950 - 0.7059x	0.59
Vigor	3.00±1.50	2.00±1.00	2.00±1.00	1.00±0.50	3.50	0.01	Y = 3.50 - 0.04x	0.42
pH	6.49±0.12	6.19±0.15	6.02±0.05	6.01±0.12	4.59	0.01	Y = -0.0322x + 6.58	0.86
Total sperm defects, %	14.20±3.15	24.72±5.01	32.89±11.01	42.89±9.40	9.39	0.01	Y = 5.115 + 0.62827x	0.78
Isolated head, %	1.00±0.50	1.20±1.00	1.80±0.50	2.00± 1.00	4.39	0.01	Y = 0.725 + 0.019x	0.51
Deformed head, %	1.00±0.50	1.20±1.00	1.75±0.50	2.00±1.00	2.45	0.01	Y = 0.725 + 0.01867x	0.50
Bent tail, %	3.00±2.00	5.00±1.50	8.00±2.00	9.00±4.00	5.65	0.01	Y = 1.00 + 0.14x	0.61
Curled tail, %	1.00±0.50	1.50±0.50	1.75±0.50	2.00±1.00	3.45	0.01	Y = 0.75 + 0.021667x	0.57

^1^All data represent the mean of 4 replicates per treatment; ^2^ CV = Coefficient of variation; ^3^ Influence of the independent variable on the dependent variable. A p<0.05 indicates a significant effect; ^4^ Math model adjusted according to the influence of the independent variable on the dependent variable.

In the second stage of analysis, the motility results (refer to Table 2) indicated that after 5 minutes, sperm subjected to the commercial extender and the samples of semen *in* natura exhibited higher motility (p<0.05) compared to those subjected to the experimental extender. This pattern persisted across the subsequent time intervals of 10, 15 and 20 minutes, with sperm treated with the commercial extender consistently demonstrating superior motility. Interestingly, at the 10- and 15-minute marks, there was a notable decline in motility observed in semen *in natura* samples, resulting in motility values very similar to those observed in sperm treated with the experimental extender.

**Table 2:** Motility (%) of rooster semen stored for 20 minutes with an experimental extender based on *Myrciaria dubia* liquid extract compared to semen *in natura* and a commercial extender.1

**Extender**	**Storage periods (minutes)**			
	**5**	**10**	**15**	**20**
Semen in natura	75.30±8.15^Aa^	68.58±10.54^Bab^	61.59±6.18^Bb^	57.75±9.46^Bc^
Experimental	66.50±8.10^Ba^	60.64±7.28^Ba^	56.30±15.50^Bab^	32.65±14.32^Cb^
Commercial	77.25±7.50^Aa^	76.30±7.69^Aa^	75.20±8.52^Aa^	70.3±5.56^Aa^
Effects	p-value			
Extender^2^	<0.001			
EP3	<0.001			
Extender x EP4	0.008			
CV5, %	4.52			

^1^All data represent the mean of 4 replicates per treatment; ^2^ Means followed by capital letters (columns) indicate a significant difference (p<0.05) between extenders; ^3^ Means followed by lowercase letters (lines) indicate a significant difference (p<0.05) between the different exposure periods (EP) evaluated; ^4^ A p-value above 0.05 demonstrates a direct influence of one factor on the result of the other and vice versa; ^5^ CV = Coefficient of variation.

In the vigor results (refer to Table 3), after 5 minutes, sperm treated with the commercial extender exhibited higher vigor (p<0.05) compared to those treated with the experimental extender. This pattern persisted across the subsequent time intervals of 10, 15, and 20 minutes, with sperm treated with the commercial extender consistently demonstrating superior vigor. However, it was observed that sperm from *in natura* samples exhibited a slower decline in vigor compared to those treated with the experimental extender.

**Table 3:** Vigor of rooster semen stored for 20 minutes with an experimental extender based on *Myrciaria dubia* liquid extract compared to semen *in natura* and a commercial extender.1

**Extender**	**Storage periods (minutes)**			
	**5**	**10**	**15**	**20**
Semen in natura	3.00±1.00^Ba^	3.00±1.00^Ba^	2.00±1.00^Bb^	2.00±1.00^Bb^
Experimental	3.00±1.50^Ba^	2.00±1.00^Cb^	2.00±1.00^Bb^	1.00±0.50^Cc^
Commercial	3.50±1.00^Aa^	3.50±1.00^Aa^	3.00±1.00^Aa^	3.00±1.00^Aa^
Effects	p-value			
Extender2	<0.001			
EP3	<0.001			
Extender x EP4	0.007			
CV5, %	5.35			

^1^All data represent the mean of 4 replicates per treatment; ^2^ Means followed by capital letters (columns) indicate a significant difference (p<0.05) between extenders; ^3^ Means followed by lowercase letters (lines) indicate a significant difference (p<0.05) between the different exposure periods (EP) evaluated ;^4^ A p-value above 0.05 demonstrates a direct influence of one factor on the result of the other and vice versa; ^5^ CV = Coefficient of variation.

In the pH analysis (refer to Table 4), after 5 minutes, samples treated with the experimental extender exhibited lower pH values (p< 0.05). Samples from semen *in natura* and those treated with the commercial extender displayed a similar trend, with pH values above 7, indicating a basic pH.

**Table 4:** The pH of rooster semen stored for 20 minutes with an experimental extender based on *Myrciaria dubia* liquid extract compared to semen *in natura* and a commercial extender.1

**Extender**	**Storage periods (minutes)**			
	**5**	**10**	**15**	**20**
Semen in natura	7.12±0.05^Aa^	7.02±0.03^Aa^	7.01±0.08^Aa^	6.92±0.06^Ab^
Experimental	6.49±0.12^Ba^	6.19±0.15^Bb^	6.02±0.05^Cb^	6.01±0.12^Bb^
Commercial	7.01±0.03^Aa^	6.91±0.11^Ab^	6.85±0.05^Bb^	6.72±0.06^Ac^
Effects	p-value			
Extender2	<0.001			
EP3	<0.001			
Extender x EP4	0.002			
CV5, %	5.46			

^1^All data represent the mean of 4 replicates per treatment; ^2^ Means followed by capital letters (columns) indicate a significant difference (p<0.05) between extenders; ^3^ Means followed by lowercase letters (lines) indicate a significant difference (p<0.05) between the different exposure periods (EP) evaluated; ^4^ A p-value above 0.05 demonstrates a direct influence of one factor on the result of the other and vice versa; ^5^ CV = Coefficient of variation.

A similar trend was observed after 10, 15 and 20 minutes, with samples treated with the experimental extender consistently exhibiting lower (p<0.05) pH values. Overall, it was noted that samples with semen *in natura* and those treated with the commercial extender displayed a similar pattern, showing only a slight reduction in pH values, whereas samples treated with the experimental extender experienced a pronounced decrease in pH values.

The analysis of sperm defects (Tables 5, 6 and 7) revealed higher percentages (p<0.05) in sperm treated with the experimental extender for up to 5 minutes. Sperm from *in natura* samples and those treated with the commercial extender exhibited a similar trend. This pattern persisted across the subsequent time intervals of 10, 15, and 20 minutes, with sperm treated with the commercial extender consistently showing lower percentages (p<0.05) of sperm defects.

**Table 5:** Total sperm defects (%) of rooster semen stored for 20 minutes with an experimental extender based on *Myrciaria dubia* liquid extract compared to semen *in natura* and a commercial extender.1

**Extender**	**Storage periods (minutes)**			
	**5**	**10**	**15**	**20**
Semen in natura	12.78±4.44^ABb^	13.11±4.73^Bb^	16.22±6.36^Bab^	19.06±8.17^Ba^
Experimental	14.20±3.15^Ac^	24.72±5.01^Ab^	32.89±11.01^Aab^	42.89±9.40^Aa^
Commercial	10.63±3.88^Bb^	10.61±3.94^Bb^	10.47±3.78^Bb^	13.59±3.76^Ba^
Effects	p-value			
Extender2	<0.001			
EP3	<0.001			
Extender x EP4	<0.001			
CV5, %	8.98			

^1^All data represent the mean of 4 replicates per treatment; ^2^ Means followed by capital letters (columns) indicate a significant difference (p<0.05) between extenders; ^3^ Means followed by lowercase letters (lines) indicate a significant difference (p<0.05) between the different exposure periods (EP) evaluated; ^4^ A p-value above 0.05 demonstrates a direct influence of one factor on the result of the other and vice versa; ^5^ CV = Coefficient of variation.

**Table 6:** Defects in the sperm head (%) of rooster semen stored for 20 minutes with an experimental extender based on *Myrciaria dubia* liquid extract compared to semen *in natura* and a commercial extender.1

**Extender**	**Isolated head**				**Deformed head**			
	**Storage periods (minutes)**				**Storage periods (minutes)**			
	**5**	**10**	**15**	**20**	**5**	**10**	**15**	**20**
Semen *in natura*	0.60±0.50^AB^	1.20±1.00^A^	1.80±0.50	2.00±1.00	0.20±0.50^B^	0.40±1.00^B^	0.65±0.50^B^	1.00±0.60^B^
Experimental	1.00±0.50^A^	1.20±1.00^A^	1.80±0.50	2.00± 1.00	1.00±0.50^A^	1.20±1.00^A^	1.75±0.50^A^	2.00±1.00^A^
Commercial	0.40±0.50^B^	1.00±1.00^B^	1.80±0.50	2.00±1.00	1.00±0.50^A^	1.20±1.00^A^	1.75±0.50^A^	2.00±1.00^A^
Effects	p-value							
Extender2	0.01	0.03	0.10^ns^	0.10^ns^	0.03	0.03	0.03	0.03
EP3	0.01	0.03	0.10^ns^	0.10^ns^	0.03	0.03	0.03	0.03
Extender x EP4	0.01	0.03	0.10^ns^	0.10^ns^	0.03	0.03	0.03	0.03
CV5, %	4.90	5.00	4.60	5.10	5.00	4.30	5.10	4.50

^1^All data represent the mean of 4 replicates per treatment; ^2^ Means followed by capital letters (columns) indicate a significant difference (p<0.05) between extenders; ^3^ Means followed by lowercase letters (lines) indicate a significant difference (p<0.05) between the different exposure periods (EP) evaluated; ^4^ A p-value above 0.05 demonstrates a direct influence of one factor on the result of the other and vice versa; ^5^ CV = Coefficient of variation.

**Table 7:** Defects in the sperm head (%) of rooster semen stored for 20 minutes with an experimental extender based on *Myrciaria dubia* liquid extract compared to semen *in natura* and a commercial extender.1

**Extender**	**Bent tail**				**Curled tail**			
	**Storage periods (minutes)**				**Storage periods (minutes)**			
	**5**	**10**	**15**	**20**	**5**	**10**	**15**	**20**
Semen *in natura*	2.00±1.50^Bc^	3.50±1.50^Bb^	5.50±1.75^Bab^	7.00±3.00^Ba^	0.30±0.20^Bc^	0.50±0.25^Bb^	0.75±0.25^Bab^	1.00±1.50^ABa^
Experimental	3.00±2.00^Ac^	5.00±1.50^Ab^	8.00±2.00^Aab^	9.00±4.00^Aa^	1.00±0.50^Ac^	1.50±0.50^Ab^	1.75±0.50^Aab^	2.00±1.00^Aa^
Commercial	1.00±1.00^Cc^	1.75±0.75^Cb^	1.75±0.75^Cb^	5.00±3.00^Ca^	0.20±0.25^Bc^	0.50±0.25^Bb^	0.75±0.25^Bab^	1.00±0.50^Ba^
Effects	p-value							
Extender2	0.01	0.01	0.01	0.01	0.01	0.01	0.01	0.01
EP3	0.01	0.01	0.01	0.01	0.01	0.01	0.01	0.01
Extender x EP4	0.01	0.01	0.01	0.01	0.01	0.01	0.01	0.01
CV5, %	2.90	4.00	4.50	4.65	4.25	4.25	2.50	2.50

^1^All data represent the mean of 4 replicates per treatment; ^2^ Means followed by capital letters (columns) indicate a significant difference (p<0.05) between extenders; ^3^ Means followed by lowercase letters (lines) indicate a significant difference (p<0.05) between the different exposure periods (EP) evaluated; ^4^ A p-value above 0.05 demonstrates a direct influence of one factor on the result of the other and vice versa; ^5^ CV = Coefficient of variation.

## Discussion

Initially, we observed that the results of the preliminary quality analyses of the pure ejaculate were consistent with the standards recommended by the Brazilian College of Animal Reproduction and supported by literature references (Craft et al., 1926; Marini and Goodman, 1969; Brillard and McDaniel, 1985; Rosenstrauch et al., 1994; Kanatiyanont et al., 2012; Sonseeda et al., 2013), indicating the good reproductive quality of breeder roosters.

As noted by Makhafola et al. (2009), Jarinkovičová et al. (2012), Sonseeda et al. (2013) and Talebi et al. (2018), variations in semen quality among roosters are influenced by various factors including breed, climatic conditions, breeder age, body weight, diet, and frequency of reproduction. However, these authors also reported that breeds involved in genetic improvement programs aimed at enhancing hybrid vigor, such as the roosters utilized in this study, typically exhibit excellent reproductive characteristics.

Considering solely its individual potential, the ability of the experimental extender based on MDLE to dilute the roosters’ semen while maintaining sperm characteristics within acceptable limits for an extended duration can be deemed a noteworthy achievement for a semen extender, as indicated by various studies (Rowell and Cooper, 1960; Bellagamba et al., 2007; Rutz et al., 2007; Silva and Guerra, 2011; Lavor and Câmara, 2012; Bustani and Baiee, 2021). However, when comparing the results obtained in this study to those reported in the literature using other products for the same purpose (Sexton, 1977; Sexton and Fewlass, 1977; Łukaszewicz et al., 2020; Bustani and Baiee, 2021; Alkali et al., 2022; Taskin et al., 2022), it is evident that the experimental extender yielded less efficient than other extenders commonly used, such as egg yolk and skimmed milk (Bustani and Baiee, 2021), as well as soybean by-products (Layek et al., 2016; Bustani and Baiee, 2021).

Similarly, when comparing the efficiency of the experimental extender based on MDLE to semen *in natura* and the commercial extender in this study, all reproductive parameters assessed were impacted, with the experimental extender yielding lower results than the commercial extender. Despite the recognized abundance of nutrients and biomolecules found in MDLE, which theoretically could enhance the sperm lifespan (Zapata and Dufour, 1993; Ribeiro et al., 2016; Souza et al., 2021), the findings of this study indicate that its utilization as an extender in liquid extract form, even when combined with soy lecithin, did not elicit a positive response from sperm. It is worth noting that this outcome was consistent across all evaluated periods, with the disparity between the efficacy of the commercial extender and the inefficacy of the experimental extender becoming more pronounced with increasing sperm exposure period to these extenders.

It was also observed that the pH of the experimental extender may have adversely affected the sperm medium and consequently, sperm viability, as sperm are highly sensitive to even minimal changes in pH (Bogdonoff and Shaffner, 1954; Wilcox, 1958; Bustani and Baiee, 2021). Literature suggests that acidic pH environments are unfavorable for sperm, as they generally prefer a more basic pH environment (Rutz et al., 2007; Lavor and Câmara, 2012; Agostinho et al., 2017). An acidic pH gradually oxidizes and damages the lipid cell membrane, rendering the sperm more vulnerable to the environmental factors and more susceptible to destruction. Consequently, this diminishes their motility and vigor while increasing the percentage of dead or defective cells (Rutz et al., 2007; Lavor and Câmara, 2012), mirroring the results observed in this study.

On the other hand, MDLE was also considered as a potential constituent for a semen extender due to its abundance of antioxidant compounds, which could shield sperm from the effects of lipid peroxidation, a critical factor contributing to the decline in sperm motility and viability over time post-collection from the rooster (Rodenas et al., 2005; Khan, 2011; Jarinkovičová et al., 2012). Despite yielding results lower than the commercial extender and slightly below even semen *in natura*, the utilization of the experimental extender based on MDLE provided results deemed acceptable for up to 15 minutes based on the parameters outlined by the Brazilian College of Animal Reproduction (2013) and the literature (Rowell and Cooper, 1960; Rutz et al., 2007; Silva and Guerra, 2011; Bongalhardo, 2013; Balogun et al., 2020; Bustani and Baiee, 2021). However, beyond this timeframe, sperm quality markedly declined below these established parameters. According to Lukaszewicz et al. (2008) and Moss et al (2012), irrespective of the species, every semen sample typically contains a percentage of defective cells ranging between 20% and 30%. Nevertheless, as noted by Surai and Wishart (1996), a percentage exceeding 20% of defective cells can detrimentally affect fertility.

Osmotic pressure may also contribute to elucidating the inferior results observed with the experimental extender compared to the commercial extender and semen *in natura*. Unlike the commercial extender, which typically provides information on its pressure (355 mOsm/kg H_2_O) and pH (7.5), the experimental extender exhibited noticeable fluctuations in pH values. This suggests a potential disruption in the osmotic balance within the sperm environment, which could lead to an increased occurrence of sperm defects (Bogdonoff and Shaffner, 1954; Rowell and Cooper, 1960; Van Wambeke, 1977; Bustani and Baiee, 2021; Partyka and Nizanski, 2021), a phenomenon reflected in the observed results.

Despite the experimental extender's performance falling slightly below that of the commercial counterpart, its utilization consistently delivers results that align with or surpass the benchmarks set by existing literature (Rutz et al., 2007; Jarinkovičová et al., 2012; Partyka et al., 2012; Bongalhardo, 2013; Freitas et al., 2018). This underscores its potential as an alternative.

Roosters' semen, known for its high concentration and minimal volume, combined with its limited lifespan, necessitates solutions capable of diluting semen, augmenting fertilization potential per ejaculate, and extending the longevity of high-quality semen (Jarinkovičová et al., 2012; Partyka et al., 2012). Within the poultry industry, such innovations garner significant interest and attention, as they address critical needs and challenges faced by poultry breeders and producers (Rowell and Cooper, 1960; Bellagamba et al., 2007; Rutz et al., 2007; Silva and Guerra, 2011; Lavor and Câmara, 2012; Bustani and Baiee, 2021). Despite inherent limitations, the experimental extender fulfills these requirements, signaling its potential utility in commercial applications.

Moreover, the experimental extender's ability to consistently meet established standards underscores its promise as a commercially viable solution. Its formulation, enriched with MDLE, represents a novel approach to semen extension that warrants further exploration and refinement. Roosters' semen, renowned for its unique characteristics including high concentration and minimal volume, poses distinct challenges in the realm of reproductive technologies (Rutz et al., 2007; Jarinkovičová et al., 2012; Partyka et al., 2012; Bongalhardo, 2013; Freitas et al., 2018). Consequently, innovative solutions that can effectively address these challenges are highly sought-after within the poultry industry (Rowell and Cooper, 1960; Bellagamba et al., 2007; Silva and Guerra, 2011; Lavor and Câmara, 2012; Bustani and Baiee, 2021). While the experimental extender may exhibit slightly lower efficacy compared to established commercial formulations, its consistent ability to meet or surpass industry standards highlights its potential as a valuable addition to the poultry breeder's toolkit.

## Conclusion

The findings of the present study suggest that MDLE holds promise as a primary component in rooster semen extender, capable of preserving sperm characteristics within acceptable thresholds for up to 15 minutes at room temperature. However, it is worth noting that the experimental extender exhibited lower efficacy compared to its commercial counterpart, established in the market, in sustaining sperm quality over the specified duration at room temperature. These results underscore the need for further testing with the MDLE-based extender to develop a formulation that achieves results equal to or even superior to commercial extenders, especially mitigating the significant decrease in pH, i.e., the acidifying the environment to the sperm.

Thus, we suggest future research should focus on validating the antioxidative effects of MDLE-diluted semen and exploring its potential to enhance fertilizing capacity compared to semen in natura. It is crucial to conduct fertility tests to confirm the actual fertilizing capacity of MDLE-treated semen. While our initial findings suggest that MDLE may preserve sperm characteristics for a short duration, further studies are needed to optimize the formulation and fully realize its potential as a viable alternative to commercial extenders. These future investigations will help determine if MDLE can provide a practical and effective solution for poultry producers, especially those with limited access to advanced technological resources.
